# Alteration of Manure Antibiotic Resistance Genes via Soil Fauna Is Associated with the Intestinal Microbiome

**DOI:** 10.1128/msystems.00529-22

**Published:** 2022-08-08

**Authors:** Shuai Du, Yue Zhang, Ju-Pei Shen, Hang-Wei Hu, Jie Zhang, Changlong Shu, Ji-Zheng He

**Affiliations:** a School of Geographical Sciences, Fujian Normal University, Fuzhou, China; b State Key Laboratory for Biology of Plant Diseases and Insect Pests, Institute of Plant Protection, Chinese Academy of Agricultural Sciences, Beijing, China; c College of Resources and Environment, Huazhong Agricultural University, Wuhan, China; d Cangzhou Academy of Agricultural and Forestry Sciences, Hebei Key Laboratory of Soil Entomology, Cangzhou, China; Marquette University

**Keywords:** antibiotic resistance genes, bioconversion, gut microbiome, microbial interaction, pathogenicity-related genes, shotgun metagenome sequencing

## Abstract

Livestock wastes contain high levels of antibiotic resistance genes (ARGs) and a variety of human-related pathogens. Bioconversion of livestock manure using larvae of the beetle Protaetia brevitarsis is an effective technique for waste reduction and value creation; however, the fate of manure ARGs during gut passage and interaction with the gut microbiome of *P. brevitarsis* remains unclear. To investigate this, we fed *P. brevitarsis* with dry chicken manure for 6 days and measured bacterial community dynamics and ARG abundance and diversity along the *P. brevitarsis* gut tract using high-throughput quantitative PCR and metagenomics approaches. The diversity of ARGs was significantly lower in larval midgut, hindgut, and frass than in raw chicken manure, and around 80% of pathogenicity-related genes (PRGs) exhibited reduced abundance. Network analysis demonstrated that *Bacteroidetes* and *Firmicutes* were the key bacterial phyla associated with ARG reduction. Metagenomic analysis further indicated that ARGs, mobile genetic elements (MGEs), and PRGs were simultaneously attenuated in the hindgut, implicating a decreased likelihood for horizontal gene transfer (HGT) of ARGs among bacteria and pathogens during manure bioconversion. Our findings demonstrated that the attenuation of ARGs is strongly associated with the variation of the gut microbiome of *P. brevitarsis*, providing insights into mechanisms of risk mitigation of ARG dissemination during manure bioconversion.

**IMPORTANCE** Saprophagous fauna like the oriental edible beetle (*P. brevitarsis*) plays a fundamental role in converting organic wastes into biofertilizer. Accumulating evidence has shown that soil fauna can reduce the abundance of ARGs, although the underlying mechanism of ARG reduction is still unclear. In our previous research, we found a large reduction of ARGs in vegetable roots and leaves from frass compared with raw manure, providing a promising biofertilizer for soil-vegetable systems. Therefore, in this study, temporal dynamic changes in the microbiomes of the donor (chicken manure) and host (*P. brevitarsis*) were investigated, and we found a close association between the gut microbiome and the alteration of ARGs. These results shed new light on how the insect gut microbiome can mitigate manure-borne ARGs and provide insights into the bioconversion process via a typical member of the saprophagous fauna, *P. brevitarsis*.

## INTRODUCTION

The modern livestock industry is characterized by large-scale intensive farms and massive waste production, due to the growing demand for food and proteins from global population growth. Animal excrement is traditionally used as fertilizers in agroecosystems, but considerable concerns for its safety in agricultural application have recently been raised, owing to the intensive use of antibiotics in many animal husbandry practices (1, 2). The overuse of antibiotics led to a dramatic increase in the incidence of antibiotic resistance within animal guts as well as excrement, posing a potential risk of the migration of antibiotic resistance genes (ARGs) into pathogenic bacteria through horizontal gene transfer (HGT) (3, 4). It is therefore imperative to mitigate the spread of manure-derived ARGs in environmental settings, which requires great efforts devoted to the development of effective approaches to reduce ARG loadings before land application.

Traditional anaerobic digestion and thermophilic composting have demonstrated various degrees of reduction of the diversity and abundance of ARGs in livestock waste (5–8). Meanwhile, bioconversion technology using saprophagous animals is another major strategy for recycling nutrients and has shown promising potential in reducing the abundance of manure-borne ARGs (9–11). The gut microbiome is critical to the host physiology and coevolution of insects, owing to its pivotal functions in digestion, detoxification, and pathogen resistance (12). The intestinal microbiome may respond actively to selection pressures, compete with invading organisms, and form multiple associations among its members, thereby providing health benefits to the host (13). Previous studies have characterized microbial changes across different diets (14), ages (15), and habitats (16) for various insect species, particularly scarabs, but few studies have attempted to identify the indigenous gut microbes that are metabolically active and that suppress the growth of manure ARG-carrying bacteria during the digestion process.

Protaetia brevitarsis is a species of beetle in the family Scarabaeidae whose larva is saprophagous and feeds on a wide range of organic resources, primarily due to the enormous microbial diversity and functional compartmentalization of its digestive tract (14, 17–19). The mechanism of manure bioconversion via saprophagous animals is mostly associated with the role of the gut microbiome (9). Intestinal bacteria are dominated by *Proteobacteria*, *Firmicutes*, *Actinobacteria*, and *Bacteroidetes*, but their proportions vary with species type (20), foods (21), and the surrounding environment (22). Distinct gut characteristics were found in different functional compartments, such as enzyme expression, body size, and pH. For example, the midgut of *P. brevitarsis* occupies most of the body size with a highly alkaline pH, and the hindgut contains the majority of the intestinal microbiome with a moderately alkaline pH (14). These biotic and abiotic factors greatly aid waste fermentation (23) and refine the microbial community along the digestion process (17). Currently, we have limited knowledge of the relationships between microbial community succession and the changes in the antibiotic resistome derived from livestock waste in the *P. brevitarsis* gut transit.

This study aimed to elucidate the variation of manure microbiome during the bioconversion process with scarab larvae and to determine the driving factors for the change of manure-borne ARGs. To achieve these aims, we fed *P. brevitarsis* with dry chicken manure for 6 days and investigated the changes of the microbial communities and antibiotic resistome of raw chicken manure (CM), larval midgut (MG), hindgut (HG) and frass (FR). In our previous research, we observed a large reduction in ARGs in the frass of *P. brevitarsis* larvae compared to chicken and swine manure, indicating larval frass as a promising biofertilizer for soil-vegetable systems (24). However, the mechanism of ARG attenuation in the digestive tract of *P. brevitarsis* larvae is still unclear. Therefore, in this study, we hypothesized that the reduction of manure-ARGs in larval frass is largely attributable to the gut microbiome of *P. brevitarsis*. Our research goal is to increase understanding of the interaction between gut and manure-derived microorganisms, with implications for designing practical options to mitigate the environmental risk of the manure-borne ARGs for the safe utilization of animal manure.

## RESULTS

### Characteristics of intestinal enzyme and pH.

**FIG 1:**
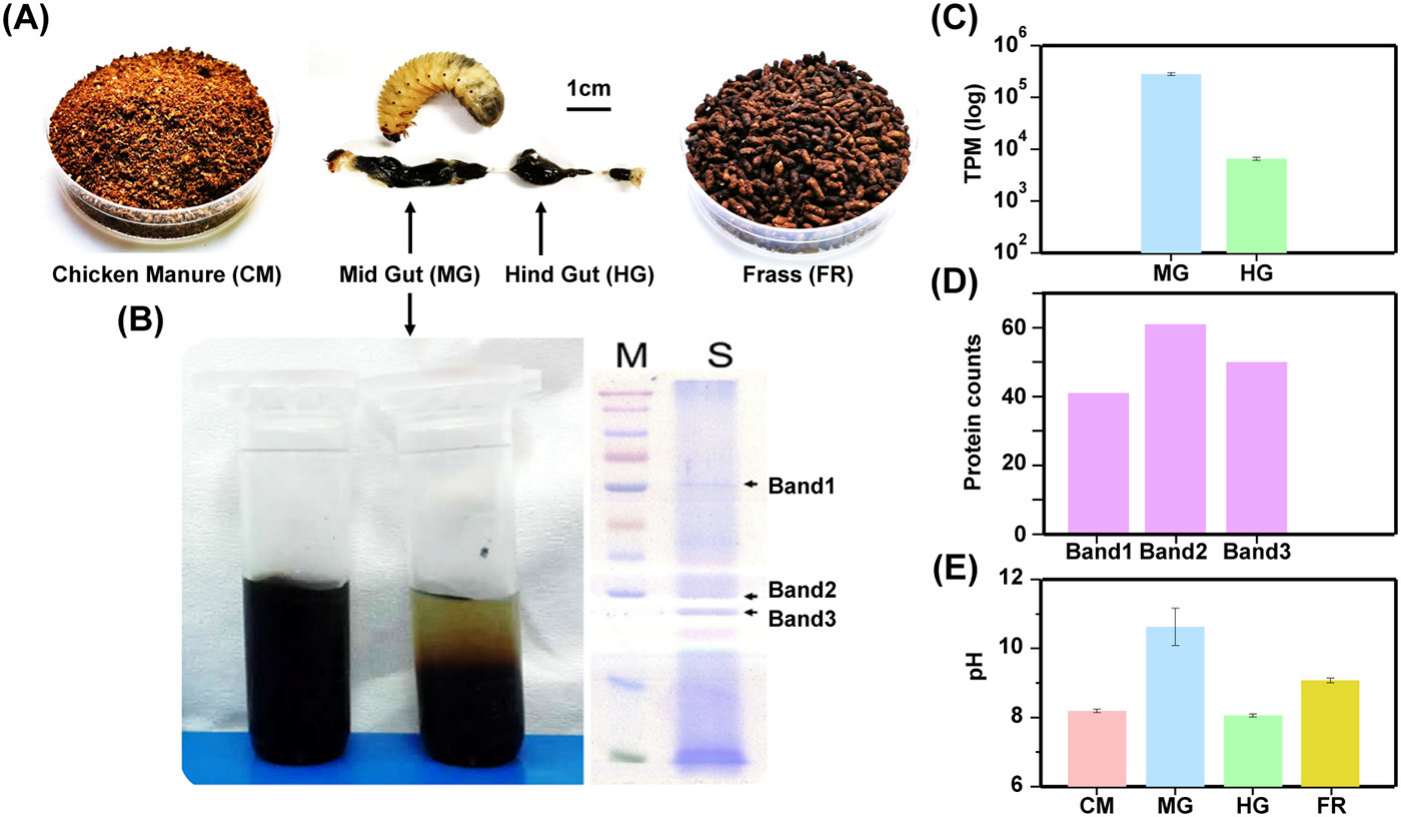
Sampling protocol and basic characteristics of the *P. brevitarsis* gut. (A) Experimental design, showing the feeding of chicken manure, digestion in the *P. brevitarsis* larval gut, and the production of frass. (B) Extraction and detection of digestive enzymes in MG. The horizontal streaks are due to the scanning process. (C) Abundance of proteolytic-enzyme-encoding genes (in TPM) in MG and HG. (D) Counts of identified protein from each band derived from digestive enzymes in MG. (E) pHs of CM, MG, HG, and FR.

Newly hatched larvae were fed with cornstalks with a moisture content of 60% until the third-instar stage of development (Fig. 1A). The digestive enzymes in the midgut and hindgut of scarab larvae were measured before feeding on chicken manure, and three main visible bands were detected in the enzyme from midgut contents using SDS-PAGE (Fig. 1B). The cumulative expression abundance of proteolytic-enzyme-encoding genes was an average of 280,886.77 (standard deviation, 18,071.55) transcripts per kilobase of exon model per million mapped reads (TPM) in the midgut and 6,519.45 (standard deviation, 397.85) TPM in the hindgut (Fig. 1C), and many proteolytic-enzyme families were retrieved from bands 1, 2, and 3 identified from the midgut enzyme gel (Fig. 1D), indicating that proteolytic enzymes were the main digestive enzymes in this system. The midgut was strongly alkaline, with a pH around 10.62 (standard deviation, 0.54), while the hindgut was marginally alkaline, with a pH of 8.06 (standard deviation, 0.04) (Fig. 1E). The information for the abundance of gut protease expression is listed in Table S1 in the supplemental material.

10.1128/msystems.00529-22.1 TABLE S1 Abundance of intestinal protease expression of *P. brevitarsis* larvae. Download Table S1, XLSX file, 0.02 MB. Copyright © 2022 Du et al. 2022 Du et al. https://creativecommons.org/licenses/by/4.0/ This content is distributed under the terms of the Creative Commons Attribution 4.0 International license.

### Diversity and abundance of ARGs and bacterial community during the digestion process.

**FIG 2:**
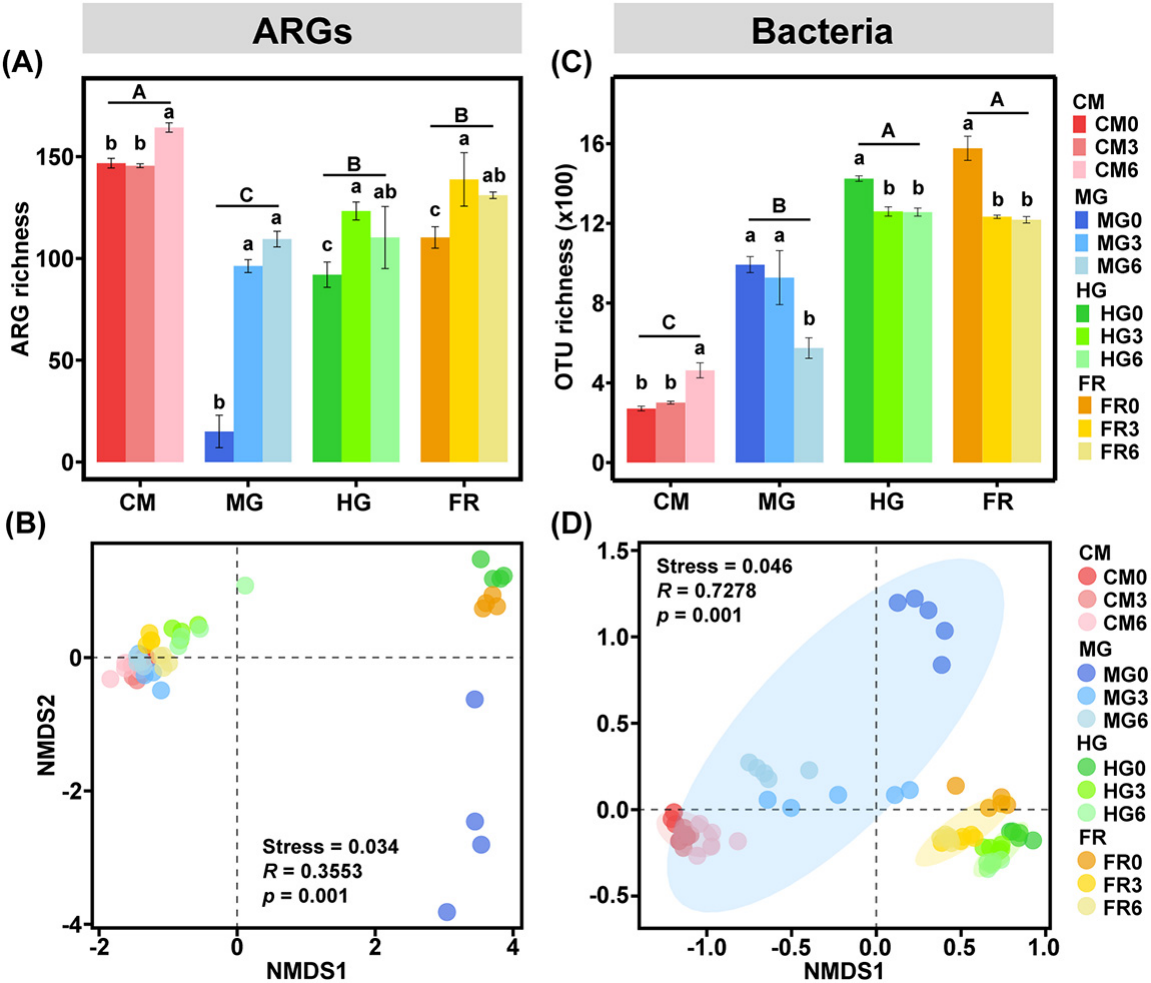
Diversity of ARGs (A and B) and bacterial communities (C and D) in CM, MG, HG, and FR using high-throughput qPCR. In panels A and C, different capital and lowercase letters indicate the significant difference between sample groups and sampling times, respectively. In panels B and D, PERMANOVA was used, and different colors of ellipses show significant differences between sample groups (95% confidence interval).

A total of 211 ARGs and 10 mobile genetic elements (MGEs) were detected in all samples using the high-throughput quantitative PCR (HT-qPCR) method and were classified into nine different categories: aminoglycoside, beta-lactamase, tetracycline, multidrug, MLSB (macrolide-lincosamide-streptogramin B), FCA (fluoroquinolone, quinolone, florfenicol, chloramphenicol, and amphenicol), sulfonamide, vancomycin, and others. The diversity of ARGs varied significantly along the digestive tract during the feeding period (analysis of variance [ANOVA], *P < *0.01) (Fig. 2A). The highest ARGs richness was recorded in CM, while the lowest in MG irrespective of feeding time. Interestingly, no significant difference in ARGs richness was found between HG and FR. Along the feeding time, ARG richness in all the samples was significantly lower at day 0 than the other two sampling times (days 3 and 6) except that in CM, indicating the influence of manure-borne ARGs (Fig. 2A). However, a decreasing trend of ARG richness was detected from day 3 to day 6 in HG and FR, while a contrasting trend was observed in CM (*P < *0.01), indicating an attenuation of ARGs through the gut. Nonmetric multidimensional scaling (NMDS) combined with permutational multivariate ANOVA (PERMANOVA) showed that ARG profiles differed significantly across samples (Adonis, *P < *0.01) (Fig. 2B). We further found that CM was significantly different from other sample types, while MG was markedly different from HG [pairwise Adonis, Pr(>F) < 0.01] (Table S2). Interestingly, ARG profiles in all the samples at day 0 were separated from each other, while those at day 3 and 6 clustered closely with CM0.

10.1128/msystems.00529-22.2 TABLE S2 Paired PERMANOVA tests on ARGs and bacterial communities. Download Table S2, XLSX file, 0.01 MB. Copyright © 2022 Du et al. 2022 Du et al. https://creativecommons.org/licenses/by/4.0/ This content is distributed under the terms of the Creative Commons Attribution 4.0 International license.

Bacterial diversity demonstrated a pattern similar to that of ARGs with the exception of CM. For the feeding treatment, the lowest bacterial operational taxonomic unit (OTU) richness was recorded in MG, while the highest was in FR (Fig. 2C). Similar to ARG richness, no significant difference in the bacterial OTU richness was found between HG and FR irrespective of feeding time. Additionally, bacterial OTU richness in all the samples except CM showed a decreasing trend with feeding time. NMDS combined with PERMANOVA analysis revealed that the bacterial communities were significantly separated among sample types irrespective of sampling time (Adonis, *P < *0.01) (Fig. 2D), while the bacterial community structures in HG and FR stayed closer. We further found that bacterial communities in CM, MG, HG, and FR were significantly different from each other [pairwise Adonis, Pr(>F) < 0.01] (Table S2). Meanwhile, sampling time had a substantial impact on bacterial community structure in the midgut (*P < *0.01), but not on other sample types. The ARGs and bacterial 16S rRNA gene copy numbers both showed similar patterns, with the highest numbers being observed in CM and the lowest in MG (Fig. S1), which are more similar to the change in ARG richness. Specifically, bacterial abundance in CM and FR showed no significant difference. With regard to feeding time, ARGs and bacterial copy numbers in CM at day 0 were both significantly lower than those at days 3 and 6, while all samples showed no significant difference between days 3 and 6.

10.1128/msystems.00529-22.4 FIG S1 Absolute abundance (gene copy numbers) of ARGs (A) and bacteria (B) in CM, MG, HG, and FR. Download FIG S1, TIF file, 0.2 MB. Copyright © 2022 Du et al. 2022 Du et al. https://creativecommons.org/licenses/by/4.0/ This content is distributed under the terms of the Creative Commons Attribution 4.0 International license.

We observed an increasing trend of shared ARGs (42, 110, and 123) between all the samples at days 0, 3, and 6, respectively (Fig. S2), and the same trend for bacterial OTUs, with 212, 287, and 502 OTUs shared among the samples at days 0, 3, and 6, respectively (Fig. S2). We further analyzed ARGs and bacterial communities using source-tracking methods (Fig. S3). The results showed that a high proportion of ARG subtypes (75% and 71% at days 3 and 6, respectively) from CM migrated into the MG and a substantial proportion (33% and 61%, respectively, at days 3 and 6) from the MG migrated into the HG. Similar to the ARG changes, a high proportion of bacterial phylotypes (79% and 85% for days 3 and 6, respectively) from CM entered the MG, while a smaller proportion (22% and 17% for days 3 and 6, respectively) from the MG entered the HG.

10.1128/msystems.00529-22.5 FIG S2 Venn diagrams showing numbers of shared and unique ARG subtypes (A to C) and bacterial OTUs (D to F) in CM, MG, HG, and FR at days 0 (A and D), 3 (B and E), and 6 (C and F). Download FIG S2, TIF file, 0.5 MB. Copyright © 2022 Du et al. 2022 Du et al. https://creativecommons.org/licenses/by/4.0/ This content is distributed under the terms of the Creative Commons Attribution 4.0 International license.

10.1128/msystems.00529-22.6 FIG S3 The source-tracking diagrams showing the variation proportion of ARGs (A and B) and bacterial OTUs (C and D) from CM to MG, HG, and FR at day 3 (A and C) and 6 (B and D). Download FIG S3, TIF file, 0.2 MB. Copyright © 2022 Du et al. 2022 Du et al. https://creativecommons.org/licenses/by/4.0/ This content is distributed under the terms of the Creative Commons Attribution 4.0 International license.

### Network analysis of ARGs and bacterial community.

**FIG 3:**
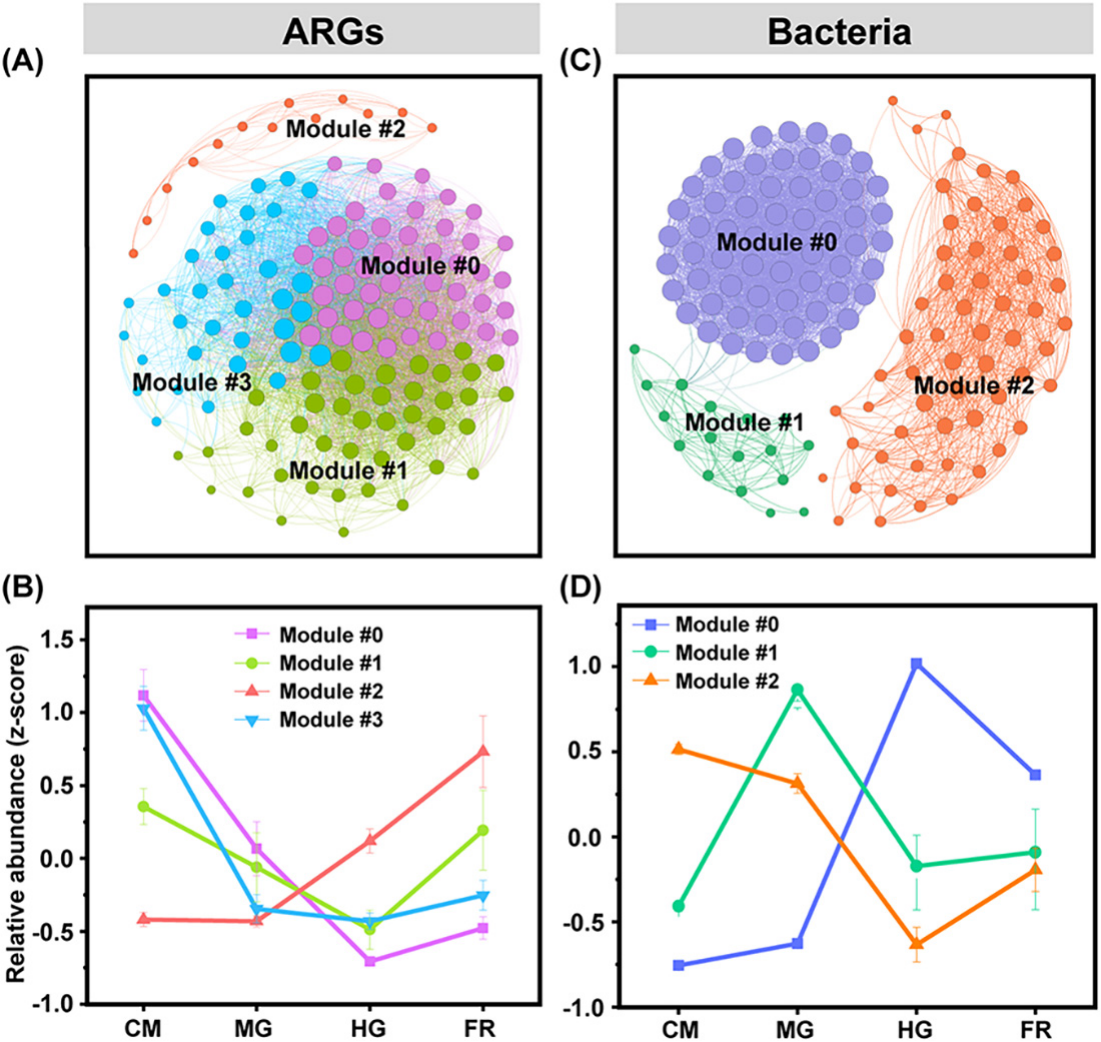
Co-occurrence network analysis of ARGs (A) and bacterial communities (C) and relative abundances of modules (B and D). In panels A and C, each node represents an ARG subtype or bacterial OTU. The different colors of the nodes represent the different ecological modules. The relative abundance of each module was normalized by the z-score method (i.e., the mean of all values is 0 and the standard deviation is 1).

The correlation network based on the relative abundance of ARGs resulted in four modules across all the samples (Fig. 3A). ARG modules 0 and 1 harbored diverse ARG types, while modules 2 and 3 had fewer ARG types (Fig. S4). For example, module 2 was mainly composed of ARGs conferring resistance to tetracycline (Fig. S4A), and the relative abundance showed an increasing trend along the digestion transit (Fig. 3B). Module 3 harbored ARGs mainly conferring resistance to MLSB and aminoglycoside (Fig. S4A), showing a decreasing trend along the digestion transit (Fig. 3B). Module 0 showed a trend similar to that of module 3, while module 1 decreased along the tract of CM-MG-HG and increased in FR (Fig. 3B).

10.1128/msystems.00529-22.7 FIG S4 Network ecological modules of ARGs types (A), bacterial community compositions at the phylum level (B), and positive and negative correlations between bacterial and ARG modules (C and D). Different colors of nodes show different bacterial phyla in the networks. Green and red edges show positive and negative correlations between bacterial OTUs and ARGs, respectively. Download FIG S4, TIF file, 1.2 MB. Copyright © 2022 Du et al. 2022 Du et al. https://creativecommons.org/licenses/by/4.0/ This content is distributed under the terms of the Creative Commons Attribution 4.0 International license.

A bacterial correlation network was built based on the relative abundance and was divided into three ecological modules (Fig. 3C). Modules 0 and 2 contributed greatly to the changes of total bacterial community structure, containing 80% of the bacterial OTUs. The dominant bacterial phyla were *Bacteroidetes* (63.0%), and *Firmicutes* (22.3%) in module 0, while *Firmicutes* (mean, 55%), *Proteobacteria* (mean, 20%), and *Actinobacteria* (mean, 20%) were dominant in modules 1 and 2 (Fig. S4B). The total relative abundance of bacterial module 0 increased along the gut transit from CM to FR, while that of module 2 decreased (Fig. 3D).

### Interaction and co-occurrence analysis between ARGs and the bacterial community.

**FIG 4:**
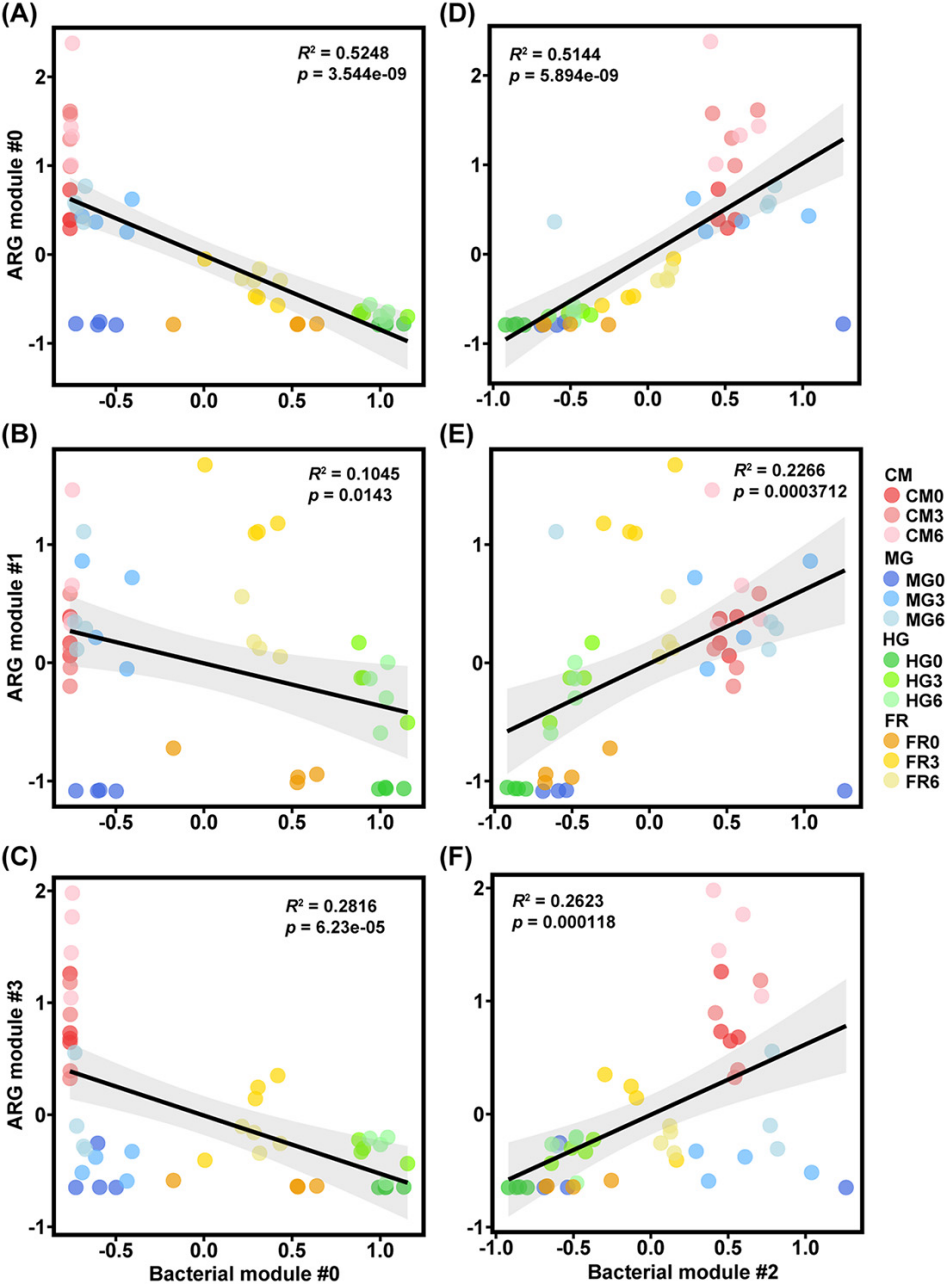
Linear regression demonstrates correlations between bacterial and ARG network modules. (A to C) Bacterial module 0 and ARG modules 0, 1, and 3. (D to F) Bacterial module 2 and ARG modules 0, 1, and 3. These ecological modules refer to network results shown in Fig. 3. Pearson’s correlations between the relative abundance (z-score) of bacterial modules and those of ARG modules were calculated. The *R*^2^ values were used to explain the linear regressions between bacterial modules and ARG modules, and the *P* values were used to test the significance. The gray area shows the 95% confidence interval.

Pearson’s correlation analysis between the main ecological modules of ARGs (modules 0, 1, and 3) and bacterial community (modules 0 and 2) was carried out. Interestingly, ARG modules 0, 1, and 3 had significantly negative correlations with bacterial module 0 but significantly positive correlations with bacterial module 2 (Fig. 4). Network analysis between main bacterial modules and ARGs further revealed that the phyla *Bacteroidetes* and *Firmicutes* in bacterial module 0 had strong negative interactions with ARGs, while the phylum *Firmicutes* in bacterial module 2 had strong positive interactions with ARGs (Fig. S4C and D), indicating their roles as the keystone species modulating the occurrence of ARGs. We also performed the Mantel test and found that ARG assemblages were significantly positively correlated with bacterial communities (*r *= 0.74, *P < *0.01). Procrustes analysis further supported the tight associations between ARGs and bacterial communities (*M*^2^ = 0.31, *P < *0.01).

### Metagenomic attributes of ARGs, MGEs, and bacterial PRGs.

**FIG 5:**
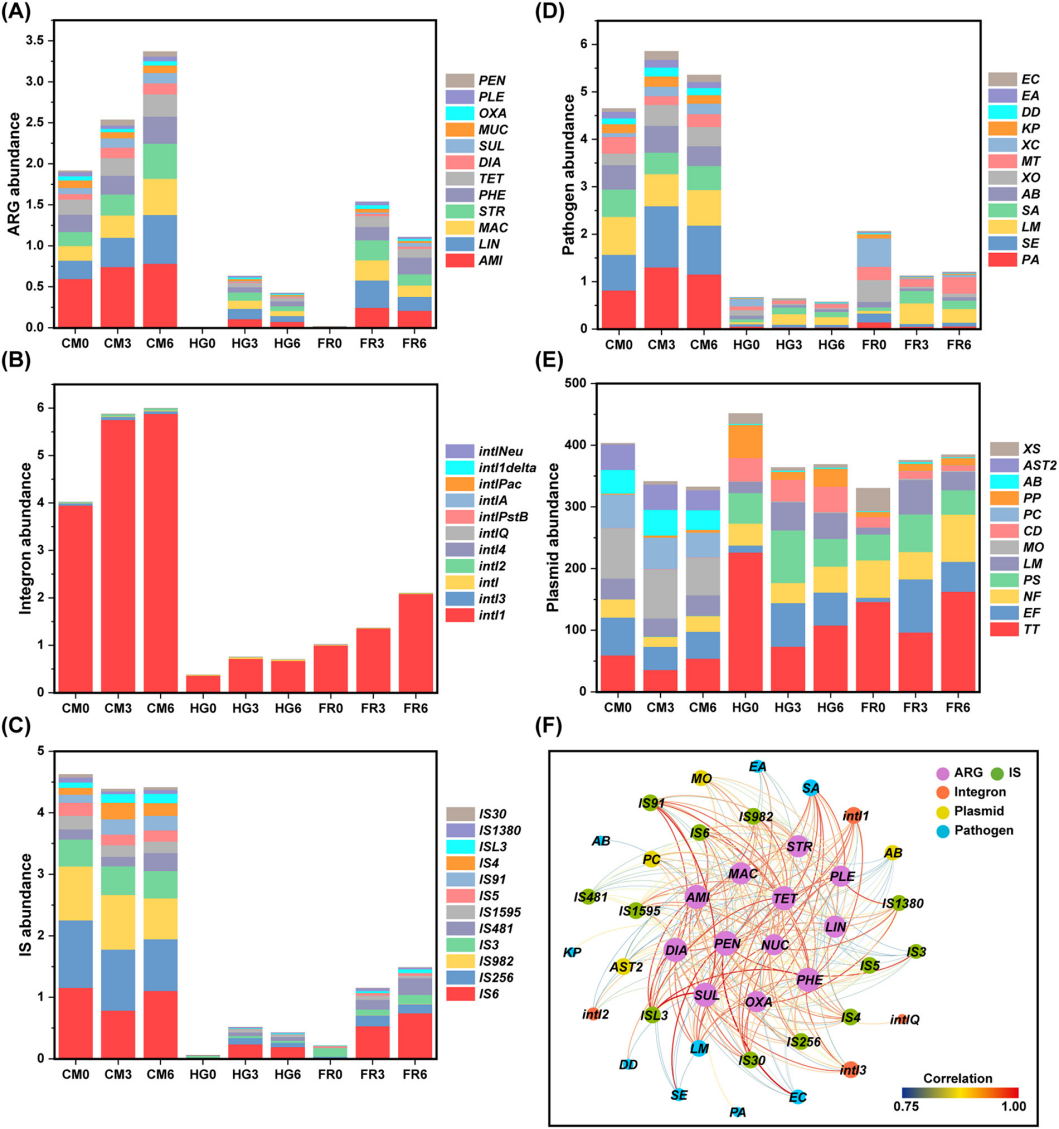
Metagenomic attributes of ARGs (A), MGE genetic markers, including integrons (B), ISs (C), and plasmids (D), and PRGs (E) and correlations between ARGs, MGEs, and PRGs (F). The units of the *y* axes are TPM (10^3^). AMI, aminoglycoside; LIN, lincosamide; MAC, macrolide; STR, streptogramin; PHE, phenicol; TET, tetracycline; DIA, diaminopyrimidine; SUL, sulfonamide; NUC, nucleoside; OXA, oxazolidinone; PLE, pleuromutilin; PEN, penam; MO, Moraxella osloensis; PC, Psychrobacter cryohalolentis; AB, Acinetobacter baumannii; AST2, uncultured bacterium AST2; PA, Pseudomonas aeruginosa; SE, Salmonella enterica; LM, Listeria monocytogenes; SA, Staphylococcus aureus; KP, Klebsiella pneumoniae; DD, Dickeya dadantii; EA, Erwinia amylovora; EC, Escherichia coli.

To verify the differences in microbial changes, 9 metagenomes for CM, HG, and FR samples were constructed, while the MG was eliminated due to low biomass. The ARGs and the genetic markers for MGEs in the assembled metagenomic contigs were predicted and annotated. There were 1,135 ARGs, 12,761 integrase genes, 6,696 insertion sequences (ISs), and 286,331 plasmid contigs identified (Fig. 5A to D). The relative abundance of these functional groups all showed a sharp decrease in HG and FR compared with CM. Bacterial phylogenetic analysis also indicated that CM clades were separated from the clades of HG and FR (Fig. S5). In addition, there were 28,969 pathogenicity-related genes (PRGs) belonging to 121 potential pathogens detected from the metagenome, and around 23,000 PRGs were reduced after digestion by scarab larvae (Fig. 5E).

10.1128/msystems.00529-22.8 FIG S5 Phylogenetic analysis of metagenomes profile using BlobTools with contigs assembled from sequence data derived from CM, HG, and FR. (A) Phylogenetic cluster analysis; (B) blob plot of contigs for each sample. Download FIG S5, TIF file, 1.4 MB. Copyright © 2022 Du et al. 2022 Du et al. https://creativecommons.org/licenses/by/4.0/ This content is distributed under the terms of the Creative Commons Attribution 4.0 International license.

We measured the numbers and abundances of all plasmid contigs as well as the ARGs they contained and found that nearly 85% of the detected ARG subtypes were located on plasmid contigs. The abundance of ARG-carrying plasmid contigs accounted for approximately 10% of all plasmid contigs. The abundance variations of ARG-carrying plasmids and all plasmids were inconsistent during the manure digestion process (Fig. S6). The correlation analysis between ARGs and MGEs showed that a total of 12 ISs, 4 integrase genes, and 4 plasmids retrieved from bacterial species were potentially associated with ARGs (Fig. 5F). In particular, IS91 and ISL3 had an association with the majority of ARGs, suggesting that the ISs may involve in the transmission of ARGs in the digestion process with chicken manure. It was found that 9 PRGs, especially PRGs from Staphylococcus aureus and Listeria monocytogenes, had an association with ARGs (Fig. 5F), suggesting that these species may act as vectors for ARGs. Detailed information on the abundance of ARGs, MGEs, and PRGs predicted by metagenomic analysis is provided in Table S3.

10.1128/msystems.00529-22.3 TABLE S3 Relative abundance of ARGs, integrons, insertion sequences, plasmids, and pathogens from metagenomic sequencing. Download Table S3, XLSX file, 0.01 MB. Copyright © 2022 Du et al. 2022 Du et al. https://creativecommons.org/licenses/by/4.0/ This content is distributed under the terms of the Creative Commons Attribution 4.0 International license.

10.1128/msystems.00529-22.9 FIG S6 Abundances of all plasmid contigs and plasmids containing ARGs based on metagenomic analysis. Download FIG S6, TIF file, 0.2 MB. Copyright © 2022 Du et al. 2022 Du et al. https://creativecommons.org/licenses/by/4.0/ This content is distributed under the terms of the Creative Commons Attribution 4.0 International license.

## DISCUSSION

Organic waste should be properly treated via feasible and effective techniques for agricultural waste recycling. Saprophagous fauna, such as earthworms (25), houseflies (9), black soldier flies (11), and the beetle *P. brevitarsis*, have been successfully used to process livestock waste before land application. A better understanding of the microbial interaction in the manure bioconversion process is essential to improve management strategies that support a sustainable balance between waste mitigation and reducing the risk of manure-borne pollutants like ARGs and pathogens.

**FIG 6:**
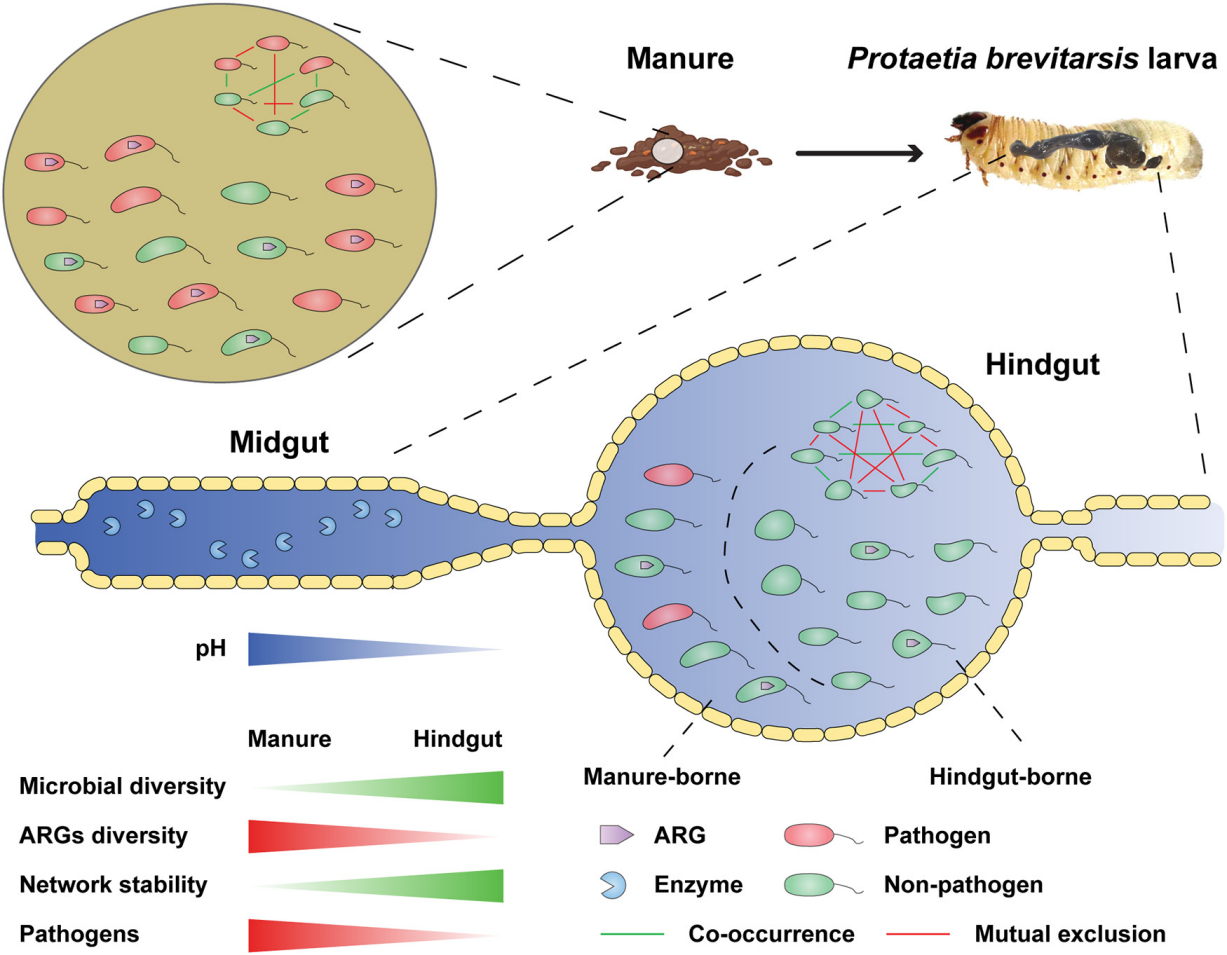
Schematic diagram of interactions between bacteria and the antibiotic resistome via *P. brevitarsis* larvae along the gut transit tract.

Dramatic changes in the abundance and diversity of gut microbiome were observed along the *P. brevitarsis* gut transect, which is in line with previous studies of other insect gut systems that utilized DNA fingerprinting techniques to assess gut microbiome dynamics (19). The unique characteristics of gut microenvironment and microbiome were the predominant drivers of ARG attenuation during manure digestion (Fig. 6). A high diversity of gut bacteria was found within the alimentary tract of *P. brevitarsis*, which has three main sections: a short crop foregut, a long midgut, and a modified extended hindgut (14). The foregut links the oral cavity and cardiac valve, providing temporary storage for food. The midgut and hindgut make up the majority of the body cavity and are separated by a muscular pyloric sphincter that regulates food flow between these two sections (26). Previous research on various scarab larvae revealed a stable structure of alimentary tract with a strongly alkaline proteolytic midgut (14, 27), which could explain the significant low abundance of bacterial community in this section. Furthermore, a relatively neutral pH in the hindgut favors the growth of certain microorganisms, which is analogous to the microorganism-rich rumen of higher mammals (28, 29). High levels of gut proteolytic enzyme gene expression obtained from gut intestine further corroborated the complex microbial community involved in the digestion of organic waste (30, 31). Altogether, gut microhabitats exert a strong selective pressure on the microbiome of the alimentary tract (32). Our metagenomic findings verified the dynamic changes of the bacterial community during manure digestion.

Manure as a rich reservoir for ARGs has substantial influence on intestinal indigenous microbiomes and antibiotic resistomes (33, 34). We fed chicken manure to *P. brevitarsis* larvae and found significant shifts of the gut microbiome and associated changes in the antibiotic resistome along the gut transit. Significantly higher richness of ARGs in the midgut was found at days 3 and 6 than at day 0. Approximately 30 ARGs, of the 73 ARGs detected in raw chicken manure, were eliminated from vermicomposting via *P. brevitarsis*. They were mostly beta-lactam resistance genes (including *bla*_CTX-M_, *bla*_MOX_, *bla*_CMY_, *bla*_PER_, and *bla*_VEB_) and multidrug resistance genes (including *acrA*, *acrB*, *acrR*, *tolC*, and *rarD*), which are generally reported to pose a high risk to human health (35, 36). These results suggested that vermicomposting via *P. brevitarsis* played a pivotal role in minimizing the potential risk of ARGs spreading to environments and humans (9, 37). The attenuation in the abundance and richness of ARGs was mainly associated with the change in gut microbiome (17). For example, ARGs conferring resistance to beta-lactams (e.g., *bla*_TEM_, *bla*_PER_, *bla*_PSE_, and *bla*_OXA_) were found to be positively associated with bacterial genera, including *Paenalcaligenes*, *Psychrobacter*, and Pseudomonas (in *Proteobacteria*). This association was further verified by the decreasing trend of bacterial module 2, which was dominated by *Firmicutes* and *Proteobacteria*. These results agree with the previous findings that the attenuated ARGs were closely related to the reduction of the bacterial order *Clostridiales* (belonging to *Firmicutes*) in the gut of housefly larvae (9).

In this study, a higher abundance of PRGs was found in the raw manure, posing a potential threat to public health by direct contact or entry into the food chain (38–40). Land application of manure introduced the manure-borne pathogenic bacteria into soils, and some of them can even survive in soil for 1 month (41). ARGs from nonpathogenic bacteria may be transferred to pathogens by HGT via MGEs, and this may render clinical antibiotics inactive. The persistence of PRGs would likely co-occurred with ARGs and potentially spread from manure to humans through the food chain, as the possibility of ARG transfer from foodborne bacteria has been verified for several strains (e.g., enterococci) *in vitro* (42). The abundance of most potential pathogens (harboring PRGs) in chicken manure was largely decreased in the hindgut and frass, indicating a great reduction of potential pathogens through larval fermentation. This result was in a good agreement with the previous research using black soldier fly (Hermetia illucens), which found a significant shift in the composition of pathogenic bacteria and attenuation in their abundance (43). This could be attributed to the fact that the larval midgut is a strongly alkaline microenvironment that devitalizes a variety of pathogenic bacteria. Pathogens and other microorganisms in biosolids have been shown to be destroyed or inactivated due to high pH (44). The alleviation of most pathogens through bioconversion would minimize the risk of dissemination of ARGs through HGT.

Changes in the bacterial community explained the largest amount of variation in ARGs and pathogens. The predominant bacterial groups obtained from the intestine were affiliated with well-known groups (e.g., *Firmicutes* and *Proteobacteria*) commonly found in other insect guts. Scarab larvae would produce inhibiting chemicals upon the entry of manure, such as proteinases, which would inhibit the growth of *Firmicutes*, *Proteobacteria*, and other bacteria (32, 45). *Firmicutes* was the main taxon associated with reduction in ARGs (46). Furthermore, high diversity of bacterial community in the hindgut can enhance host resistance to environmental stress (47–49), partly attributed to the large numbers of indigenous anaerobic bacteria dominating in the hindgut, where nearly 88% of bacterial OTUs on the 3rd or 6th feeding day were shared with larvae not fed on chicken manure. The bacterial communities in the hindgut outnumber those derived from chicken manure, thereby reducing the abundance of ARGs. Coincidentally, one study demonstrated that high microbial diversity lowers the survival and dissemination of ARGs in the gut environment (50). These findings suggest that a high level of microbial diversity can act as a biological barrier to decrease the spread of antibiotic resistance. In addition, a stable microbial community structure is also beneficial to preventing the spread of antibiotic resistance (51). In our study, a more stable bacterial community structure was found in the hindgut than in manure, based on the results of microbial networks. The metagenomic analysis revealed that ARGs were carried mainly by plasmids whose abundance decreased sharply in the *P. brevitarsis* gut, suggesting that ARG-bearing plasmids are likely to be eliminated or reduced by digestive processes during bioconversion. In summary, bioconversion of manure by *P. brevitarsis* larvae shows promise as a method to mitigate unwanted dispersal of ARGs, in particular those with high environmental risk. It has been noticed that some pathogens, such as Listeria monocytogenes, Staphylococcus aureus and Mycobacterium tuberculosis remained at a high level in the frass. Given the impact of high temperature on pathogenic bacteria (52), more studies are desirable to focus on a process coupling high-temperature composting and larval bioconversion.

### Conclusions.

In conclusion, this study experimentally demonstrates that the gut microbiome and antibiotic resistome were significantly altered after digestion of raw chicken manure, in a scenario in which members of the soil fauna are increasingly adopted to treat agricultural wastes. In particular, the different structures and microenvironments of gut compartments could contribute to the attenuation of manure-borne ARGs and PRGs. In addition, the hindgut microbiome could effectively suppress the dissemination of ARGs and PRGs by its high biodiversity and stable community structure. These findings highlight the importance of gut structure, microenvironment, and microbial communities in mitigating the dispersal of ARGs and pathogens. Future studies will concentrate on optimizing the removal efficiency of ARGs and pathogens in organic wastes by combining *P. brevitarsis* larva bioconversion with physicochemical techniques. In parallel, the frass can be used as an organic fertilizer instead of conventional livestock manure.

## MATERIALS AND METHODS

### Larva breeding and gut characteristic determination.

Scarab larvae (*Protaetia brevitarsis* Lewis) used in this study were obtained from the field as described previously by Li et al. (31). The larval digestive tract, consisting of a long, cylindrical midgut and a globose hindgut, was crosscut after removal of hemolymph with absorbent paper. The pH of the effluent gut contents was determined immediately with a Horiba ISFET pH meter. The crude digestive enzyme extract was prepared by suspending gut contents in an extraction solution (20 mM Tris-HCl, 500 mM NaCl [pH 8.0]), and the insoluble substance was discarded after centrifugation at 12,000 rpm for 10 min (4°C). The crude extract was stratified after a freeze-thawing–centrifugation process for three times to get a clarified supernatant liquid for further analysis. The clarified extracts separated by SDS-PAGE were used, and a three-band SDS-PAGE profile was obtained (Fig. 1B). The bands indicated by the arrows (bands 1 to 3) were subsequently identified using LC-MS/MS with Q Exactive (Thermo Scientific, USA), (Fig. 1B) and the resulting data were checked against the *P. brevitarsis* genome database by the Mascot software MS/MS Ion Search.

### Chicken manure feeding experiment and sampling.

Dry chicken manure was collected from a chicken farm in Cangzhou County (N 38.287, E 116.828), China, where general antibiotics, including ampicillin and erythromycin, were prescribed. The moisture content of the chicken manure was maintained at around 60% with sterile water. Before starting the chicken manure feeding experiment, third-instar *P. brevitarsis* larvae were selected and subjected to starvation for 6 h. A total of 300 *P. brevitarsis* organisms were placed in a plastic box with dimensions of 31 by 24 by 12 cm containing 0.5 kg chicken manure (dry weight) as the manure feeding treatment, and the control treatment was the same amount of chicken manure without *P. brevitarsis*. Microcosms were analyzed with five replicates for each treatment at room temperature (25°C) and sampled at days 0, 3, and 6 after first placing *P. brevitarsis* on the chicken manure. A total of 60 samples, including chicken manure (CM), midgut content (MG), hindgut content (HG), and frass (FR), were obtained for further analysis. The midgut content (MG0, MG3, and MG6), hindgut content (HG0, HG3, and HG6), and frass (FR0, FR3, and FR6) were collected from the manure feeding treatment. Chicken manure samples (CM0, CM3, and CM6) were collected from the control treatment.

Chicken manure and frass were collected and preserved at −20°C in 50-mL conical tubes for molecular analysis. For gut samples, 20 larvae were removed from the manure feeding treatment at the sampling point. The larvae were immersed in 70% alcohol for 30 s and washed three times with distilled water. The water on the larval body surface was removed with absorbent paper and kept in a sterilized plastic box for gut content sampling. The digestive tracts of the larvae were dissected, and intestines in the midgut and hindgut were collected as mentioned above. Five larvae were dissected in each procedure to extract enough gut contents, which were mixed together in 2-mL conical tubes and preserved at −80°C until further analysis.

### High-throughput sequencing and bioinformatic analyses.

The DNA was extracted with an AxyPrep multisource genomic DNA miniprep kit according to the manufacturer’s protocol and used for bacterial community sequencing and quantification, ARG profiling, and metagenomic sequencing.

The bacterial 16S rRNA gene V4 hypervariable region was amplified with the universal primer set 515F/806R (53) and sequenced using an Illumina HiSeq 2500 system (2 × 250 bp). PCR was carried out as follows: initial denaturation at 95°C for 10 min, 30 cycles at 94°C for 1 min, 55°C for 1 min, and 72°C for 1 min, plus a 10-min extension at 72°C. PCR products were purified using the AxyPrep DNA gel extraction kit (Axygen). Equal amounts of purified PCR products were mixed and extended with Illumina-specific adaptors using the TruSeq DNA PCR-free sample preparation kit (San Diego, CA, USA). The resulting libraries were sequenced using an Illumina HiSeq 2500 system (2 × 250 bp).

After filtering of low-quality reads, Illumina paired-end reads were error corrected and merged by PANDAseq. The chimeric sequences were eliminated by using the UCHIME Gold database and produced high-quality 16S rRNA gene sequences. UPARSE (USEARCH version 8.0.1517) was employed to generate the OTU table (97% identity was set as the threshold value) and determine OTU abundance. In order to count the number of sequences and species annotations of each OTU, the highest-frequency sequence was selected as the representative sequence for each OTU according to the algorithm principle. The annotation of representative OTU sequences was performed by the Ribosomal Database Project (RDP version 2.2) classifier Greengenes (version 13.8). All processes were performed using default parameters.

### Bacterial abundance and HT-qPCR for ARGs.

We used qPCR to determine bacterial 16S rRNA gene abundance on Bio-Rad iQ5 real-time PCR detection system and determined water content of each sample to calculate the dry weight ratio (DWR). Briefly, the 16S rRNA gene copy numbers were quantified using the primer set Bact1369F/Prok1492R (54) with the probe TM1389F on the Bio-Rad iQ5 real-time PCR detection system. Each 20-μL reaction mixture consisted of 10 μL Ex Taq premix (TaKaRa Biotechnology, Dalian, China), a 10 μM concentration of each primer, 1 μL DNA as the template, 1 μL probe TM1389F, and nuclease-free PCR-grade water. The plasmid DNA for generating standard curves and amplification profiles was used as described previously (55). Bacterial biomass was calculated and normalized to 16S rRNA gene copy numbers in 1 g dry matter using the following formula: bacterial biomass = 100 × 16S rRNA gene copy number/(W × DWR), where W is the weight of sample used for DNA extraction.

HT-qPCR was performed to quantify 285 ARGs on the Wafergen SmartChip real-time PCR platform (Wafergen, Fremont, CA, USA) as described previously (56). The primer sets targeted ARGs encoding resistance to eight major classes of antibiotics, including aminoglycoside, beta-lactam, FCA, MLSB, multiple drugs, sulfonamide, tetracycline and vancomycin. Three technical replicates were performed for each sample. A threshold cycle (*C_T_*) value of 31 was set as the detection limit. The relative abundances of ARGs compared to that of the 16S rRNA gene in the same HT-qPCR run were calculated according to a comparative *C_T_* method as described previously (57). The absolute abundances of ARGs were calculated as multiplying the relative abundances of ARGs by the bacterial 16S rRNA gene absolute abundance.

### Shotgun metagenomic sequencing.

Nine metagenomes of chicken manure (CM0, CM3, and CM6), larval hindgut (HG0, HG3, and HG6), and frass (FR0, FR3, and FR6) were analyzed. Midgut contents (MG) were eliminated for metagenomic analysis because of low biomass. First, paired-end libraries with 420-bp inserts were constructed using a TruSeq DNA PCR-free library prep kit, and the sequencing was performed on an Illumina HiSeq 2500 sequencer (Illumina, San Diego, CA, USA). The produced raw reads were trimmed to remove adaptor sequences, low-quality reads, and the end low-quality bases using Trimmomatic (V 0.38). The microbial community compositions were profiled by MetaPhlAn2, using the trimmed high-quality reads. All nine subset reads were assembled by MEGAHIT (version 1.2.9) and predicted by MetaProdigal (version 2.6.3). ARGs, ISs, plasmids, and PRGs were annotated by the BLAST algorithm. The genes in the assembled metagenomic contigs were predicted by MetaProdigal (version 2.6.3). The abundance of each open reading frame (ORF) was quantified with Salmon (version 1.1.0) and measured as TPM. The ARGs and insertion sequences in the metagenome were annotated by the BLAST algorithm with an E value threshold of 1e−10, a bit score of 50, and an identity of 70% using CARD (The Comprehensive Antibiotic Resistance Database [https://card.mcmaster.ca/home]) and ISfinder database (https://www-is.biotoul.fr/search.php), respectively. The plasmid sequence in the metagenome was annotated by the MEGABLAST algorithm with an E value threshold of 1e−10 using the NCBI RefSeq database (https://ftp.ncbi.nlm.nih.gov/refseq/release/plasmid/). The plasmid abundance was quantified with Salmon (version 1.1.0) and measured as TPM. The PRGs in microbial community were also analyzed; we annotated the PRGs using the PHI (pathogen-host interactions) database (http://www.phi-base.org/index.jsp) by the BLAST algorithm with an E value threshold of 1e−10, a bit score of 50, and an identity of 70%. BlobTools was used to visualize the metagenome taxonomic partitioning of different samples (58).

### Statistical analyses.

One-way ANOVA was performed to detect the differences in the diversity and abundance of bacteria as well as ARGs between CM, MG, HG, and FR using SPSS 22 (IBM, Armonk, NY, USA) with a statistically significant value for *P* of <0.05. The changes of bacterial community compositions as well as ARGs during the chicken manure digestion were calculated by NMDS ordinations based on the Bray-Curtis dissimilarity distances and PERMANOVA with 999 permutations. The differences in bacterial communities as well as ARGs between every two groups were further tested via the pairwiseAdonis package in R. Regression analyses were performed to decipher the correlations between bacterial community and ARGs, and linear models were used to estimate the curve fitting with adjusted *P* values of <0.05. These analyses were performed using the vegan package (58) and plotted using the ggplot2 package in the R platform (59). We used Venn diagrams to depicture the shared and unique bacterial OTUs and ARGs in CM, MG, HG, and FR (http://bioinformatics.psb.ugent.be/webtools/Venn/) and Source Tracker (v.1.0) (60) based on a Bayesian approach, to estimate the sources of the bacterial communities and ARGs in guts and frass.

We constructed a co-occurrence network to explore the potential interactions among microbial communities and antibiotic resistomes, calculated pairwise Spearman correlations using the WGCNA package (61), and visualized networks in Gephi (62). All pairwise Spearman correlations were calculated, and those with an absolute value of Spearman’s rho of >0.6 were retained. All *P* values were adjusted by the Benjamini-Hochberg false discovery rate (FDR) test with a 0.01 cutoff. We used Gephi (default parameters) to identify the ecological modules of microbial interactions. The relative abundance of each module was calculated by averaging the normalized relative abundances (z-score) of the phylotypes within each module. We use the following formula to perform a z-score normalization on every value in a data set: new value = (*x* − μ)/σ, where *x* represents the original value, μ represents the mean of data, and σ represents the standard deviation of data.

### Data availability.

Bacterial sequences have been submitted to the NCBI database with the accession number PRJNA742600. Metagenomic data have been submitted with the accession numbers SRR14211378, SRR14212118, SRR14224883, SRR14293692, SRR14297457, SRR14297764, SRR14298063, SRR14298725, and SRR14300306.
