# Supplementary figures and images for: Alteration of Manure Antibiotic Resistance Genes via Soil Fauna Is Associated with the Intestinal Microbiome
Source: mSystems. 2022 Aug 8;7(4):e00529-22. doi: 10.1128/msystems.00529-22 (PMC9426575; doi:10.1128/msystems.00529-22)

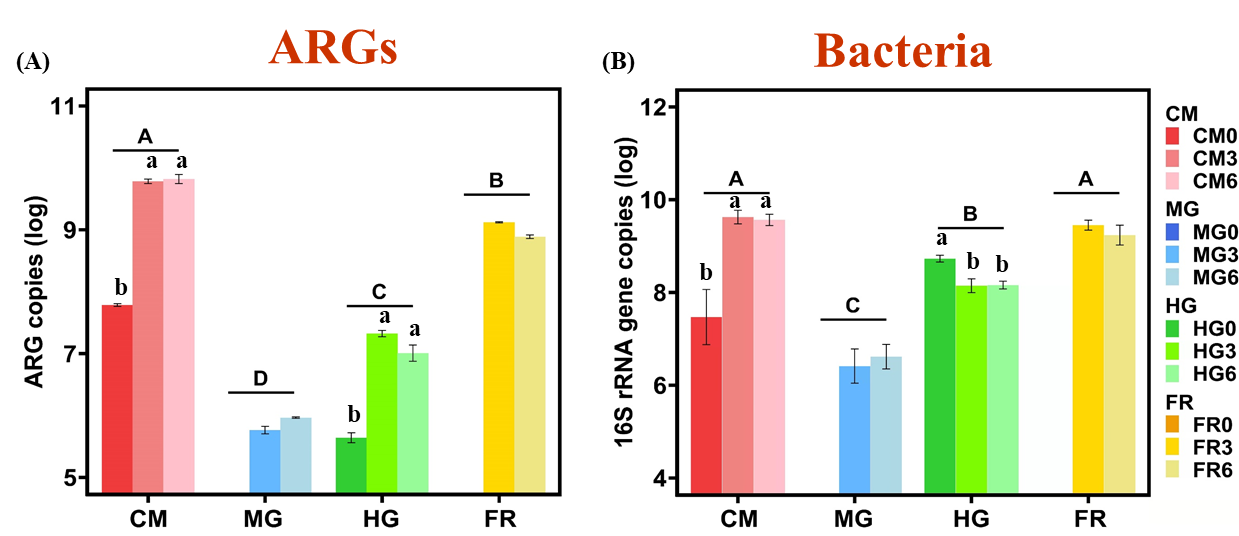

Supplement: FIG S1 [file msystems.00529-22-s0004.tif]

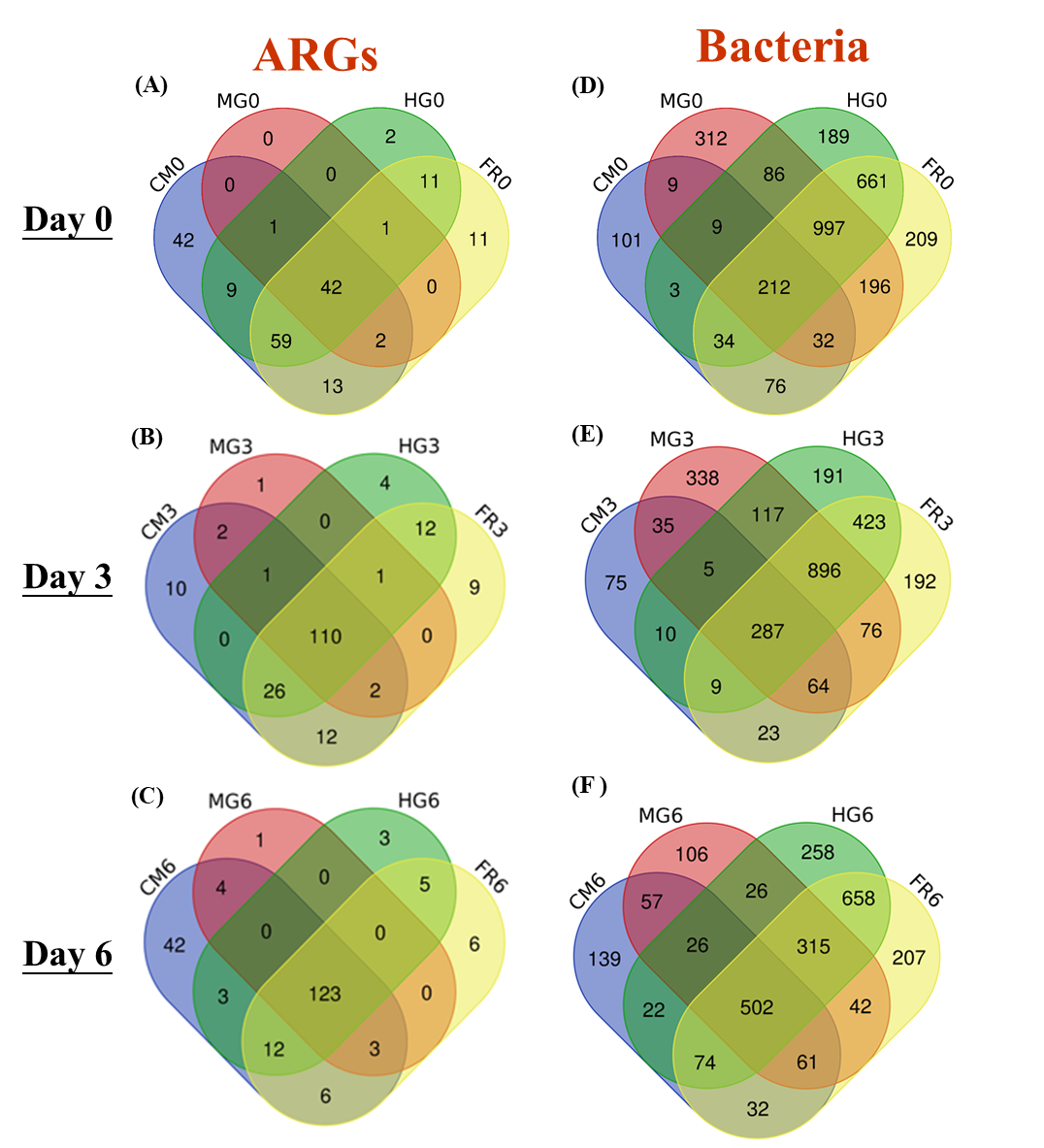

Supplement: FIG S2 [file msystems.00529-22-s0005.tif]

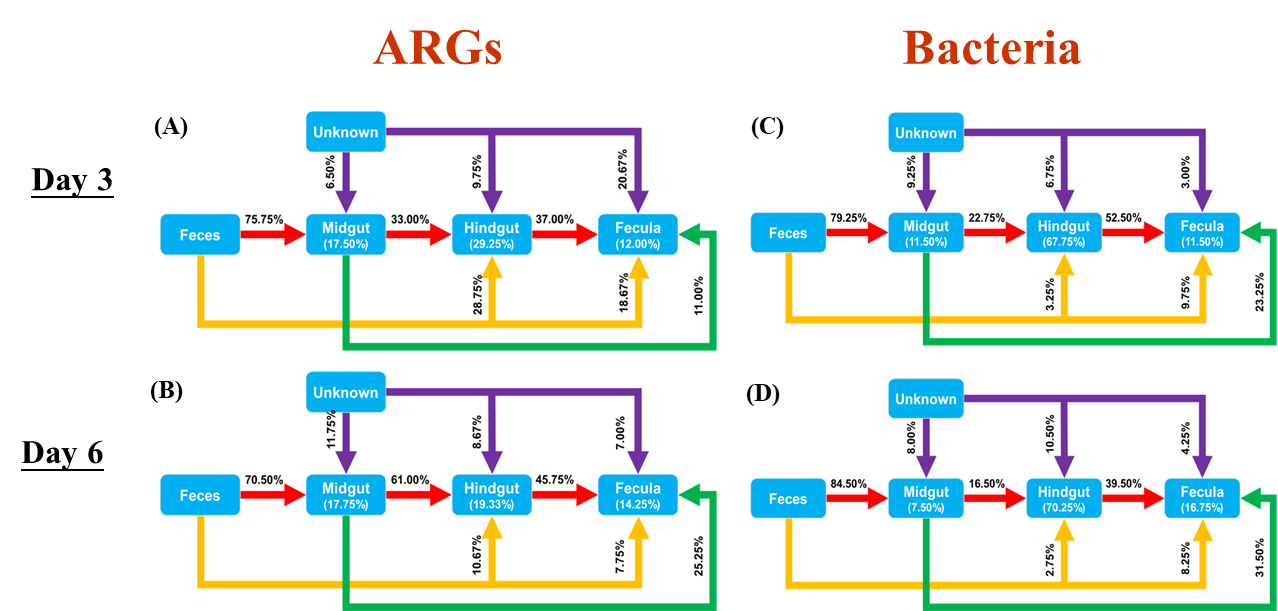

Supplement: FIG S3 [file msystems.00529-22-s0006.tif]

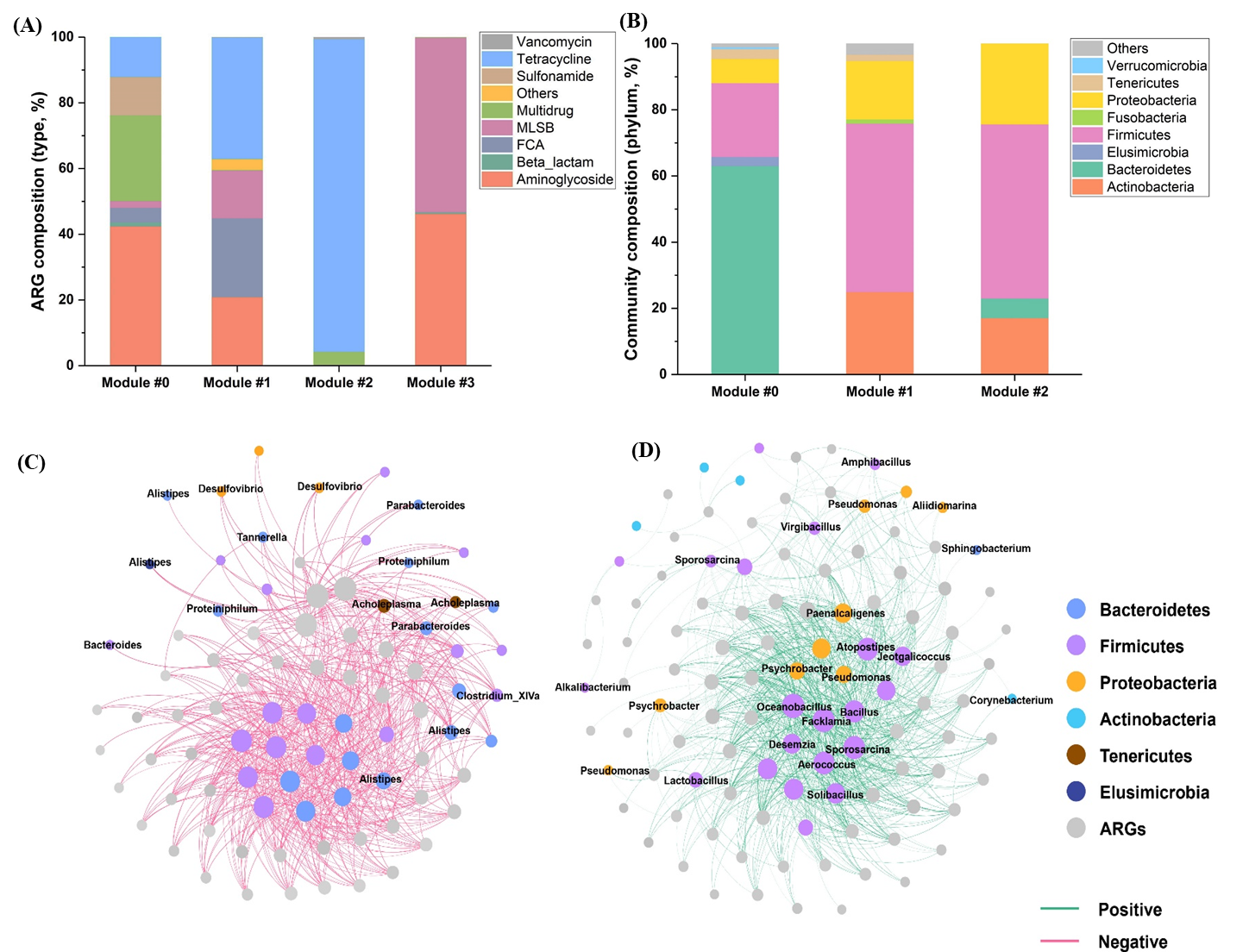

Supplement: FIG S4 [file msystems.00529-22-s0007.tif]

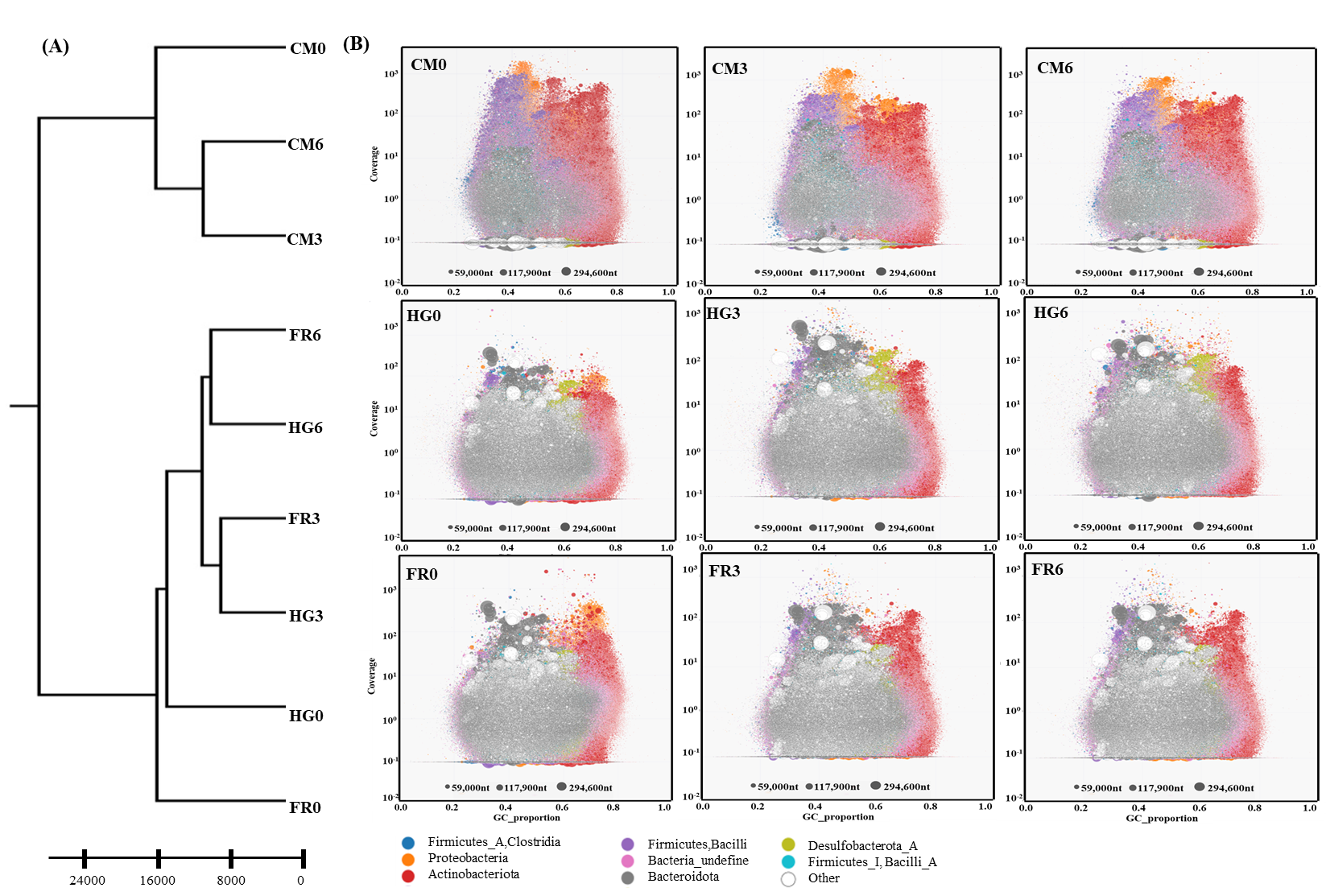

Supplement: FIG S5 [file msystems.00529-22-s0008.tif]

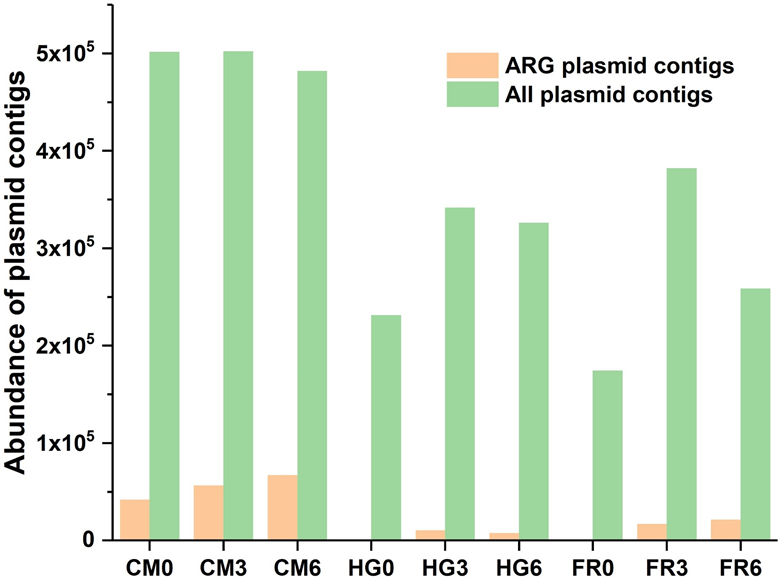

Supplement: FIG S6 [file msystems.00529-22-s0009.tif]
